# WeChat assisted electronic symptom measurement for patients with adenomyosis

**DOI:** 10.1186/s12911-024-02570-8

**Published:** 2024-06-17

**Authors:** Wei Xu, Xin Zhang, Fan Xu, Yuan Yuan, Ying Tang, Qiuling Shi

**Affiliations:** 1 College of Public Health, Chongqing Medical University, Chongqing, China; 2 State Key Laboratory of Ultrasound in Medicine and Engineering, College of Biomedical Engineering, Chongqing Medical University, Chongqing, China; 3 Department of Obstetrics and Gynecology, Nanchong Central Hospital, Second Clinical Medical College, North Sichuan Medical University, Nanchong, Sichuan China; 4 Department of Obstetrics and Gynecology, Affiliated Hospital of Southwest Medical University, Luzhou, Sichuan China

**Keywords:** Electronic PRO-based symptom management, Adenomyosis, WeChat group-based doctor-patient communication, Patient-reported outcomes, Validity, Symptom burden

## Abstract

**Purpose:**

Symptom assessment is central to appropriate adenomyosis management. Using a WeChat mini-program-based portal, we aimed to establish a valid symptom assessment scale of adenomyosis (AM-SAS) to precisely and timely identify needs of symptom management and ultimately, to alert disease recurrence.

**Methods:**

A combination of intensive interviews of patients with adenomyosis and natural language processing on WeChat clinician-patient group communication was used to generate a pool of symptom items-related to adenomyosis. An expert panel shortened the list to form the provisional AM-SAS. The AM-SAS was built in a Wechat mini-programmer and sent to patients to exam the psychotically validity and clinical applicability through classic test theory and item response theory.

**Results:**

Total 338 patients with adenomyosis (29 for interview, 179 for development, and 130 for external validation) and 86 gynecologists were included. The over 90% compliance to the WeChat-based symptom evaluate. The AM-SAS demonstrated the uni-dimensionality through Rasch analysis, good internal consistency (all Cronbach’s alphas above 0.8), and test-retest reliability (intraclass correlation coefficients ranging from 0.65 to 0.84). Differences symptom severity score between patients in the anemic and normal hemoglobin groups (3.04 ± 3.17 vs. 5.68 ± 3.41, *P* < 0.001). In external validation, AM-SAS successfully detected differences in symptom burden and physical status between those with or without relapse.

**Conclusion:**

Electronic PRO-based AM-SAS is a valuable instrument for monitoring AM-related symptoms. As an outcome measure of multiple symptoms in clinical trials, the AM-SAS may identify patients who need extensive care after discharge and capture significant beneficial changes of patients may have been overlooked.

**Trial registration:**

This trial was approved by the institutional review board of the Chongqing Medical University and three participating hospitals (Medical Ethics Committee of Nanchong Central Hospital, Medical Ethics Committee of Affiliated Hospital of Southwest Medical University, and Medical Ethics Committee of Haifu Hospital) and registered in the Chinese Clinical Trial Registry (registration number ChiCTR2000038590), date of registration was 26/10/2020.

**Supplementary Information:**

The online version contains supplementary material available at 10.1186/s12911-024-02570-8.

## Introduction

Adenomyosis is a common benign gynecologic condition characterized by the invasion or diffusion of endometrial glands and stroma into the myometrium of patients of childbearing age [1]. The prevalence of adenomyosis increases with age, reaching a peak of 32% in patients aged 40–49 years with infertility [2]. Adenomyosis is being increasingly diagnosed in patients [3]. A magnetic resonance imaging study of patients younger than 42 years with symptomatic benign gynecological conditions showed that isolated diffuse adenomyosis occurred in one-third of the study population (34.6%) [4]. While a definitive diagnosis through histology remains the gold standard, Several studies have shown that transvaginal ultrasound (TVUS) can be considered the first-line imaging modality for studying adenomyosis because it is non-invasive, sensitive and specific [5, 6]. 

Typical symptoms of adenomyosis include menorrhagia, pelvic pain, menorrhagia, bloating, infertility, and dysmenorrhea [7, 8]. Except for the option of hysterectomy, which is considered the gold standard for patients who have completed childbearing, here are no drugs or standard procedures have been approved by the United States (US) Food and Drug Administration (FDA) for the treatment of adenomyosis [8]. For reproductive-aged patients and those wishing to retain their uterus, conservative therapy could be offered [9], which requires effective long-term management to maintain the treatment effect and delay symptom recurrence.

Some studies revealed that dysmenorrhea or subjective symptoms recurred in 26–50% of patients during the first year of conservative therapy without close monitoring and management [10–12]. Currently, systematic, standard, and effective long-term management measures and tools are lacking for patients with adenomyosis. Thus, an effective symptom management tool is essential to accelerate the precise and timely identification of care needs for symptom assessment and diagnosis of recurrent disease [1, 13]. 

Patient-reported outcomes (PROs) are self-assessments that directly measure symptoms and functional status [14] and engage patients as active participants, which may improve the experience, efficiency, and outcomes of care [15, 16]. Symptom assessment is the foundation for the diagnosis of adenomyosis and forms the principles of effective management strategies in the disease process. Thus, an accurate symptom description is crucial for management with the aim of improving the quality of life in patients with adenomyosis. According to a targeted literature review, no PRO measures for adenomyosis have been developed, and information regarding the specific signs, symptoms, and impact experienced by patients with adenomyosis is limited. The measure most used to evaluate adenomyosis-related symptoms, the uterine fibroid symptom and health-related quality of life (UFS-QOL) [17], does not delve into adenomyosis domains. Adenomyosis may have its own specific symptoms that differ from those of fibroid, in particular, pain (abdominal-, pelvic-, low back- and menstrual pain); [8] thus, the UFS-QOL may lack the sensitivity to capture pre- to postoperative symptom changes and burden. Given the increasing emphasis on patient-centered outcomes [18], a validated PRO measure could benefit symptomatic improvement for extensive regulatory care [8]. 

Internet penetration reached 54% at the end of 2018 in China, making social media an important source of clinical monitoring [19]. Surgeons can use social media to gather unbiased information of patients’ experiences to inform clinical conversation and guide clinical practice [20]. With the most active users of any social media platform in China, WeChat has become the preferred platform for public announcements and is widely used in medicine and nursing [21]. WeChat mini program is a communication service application have been successfully use to timely and effective symptom management [22]. 

WeChat is downloaded to almost every Chinese mobile phone. Therefore, WeChat records contain tremendous free and semi-structured data of doctor-patient communications. Importantly, WeChat in real world to generate more representative symptom that meaningful to patients compare to interview. Natural Language Processing (NLP) uses algorithms to automatically extract and construct relevant clinical information from unstructured data [23]. Compared with manual identification, the data automatically extracted by NLP could ensure efficient and accurate identification of patient-reported symptoms [24]. Therefore, we used the WeChat data as the supplementary based on the conventional concept-elicitation interviews process to ensure the accuracy and integrality of specific symptoms. At the same time, we built in a WeChat mini-programmer and sent to patients to exam the psychometrical validity and clinical applicability.

Our study aimed to establish a valid symptom assessment instrument base on WeChat-min-program to precisely and timely identify needs of symptom management and ultimately, to alert disease recurrence. Firstly, we explore the feasibility of developing reliable assessment scales in WeChat mini-programmer through the use of natural language processing on un-structed WeChat data. Secondly, we aimed to provide psychometric evidence supporting the reliability and validity of scales from a novel PRO tool specific to core symptom assessment of adenomyosis (AM-SAS) in WeChat mini-programmer.

## Materials and methods

**Fig. 1:**
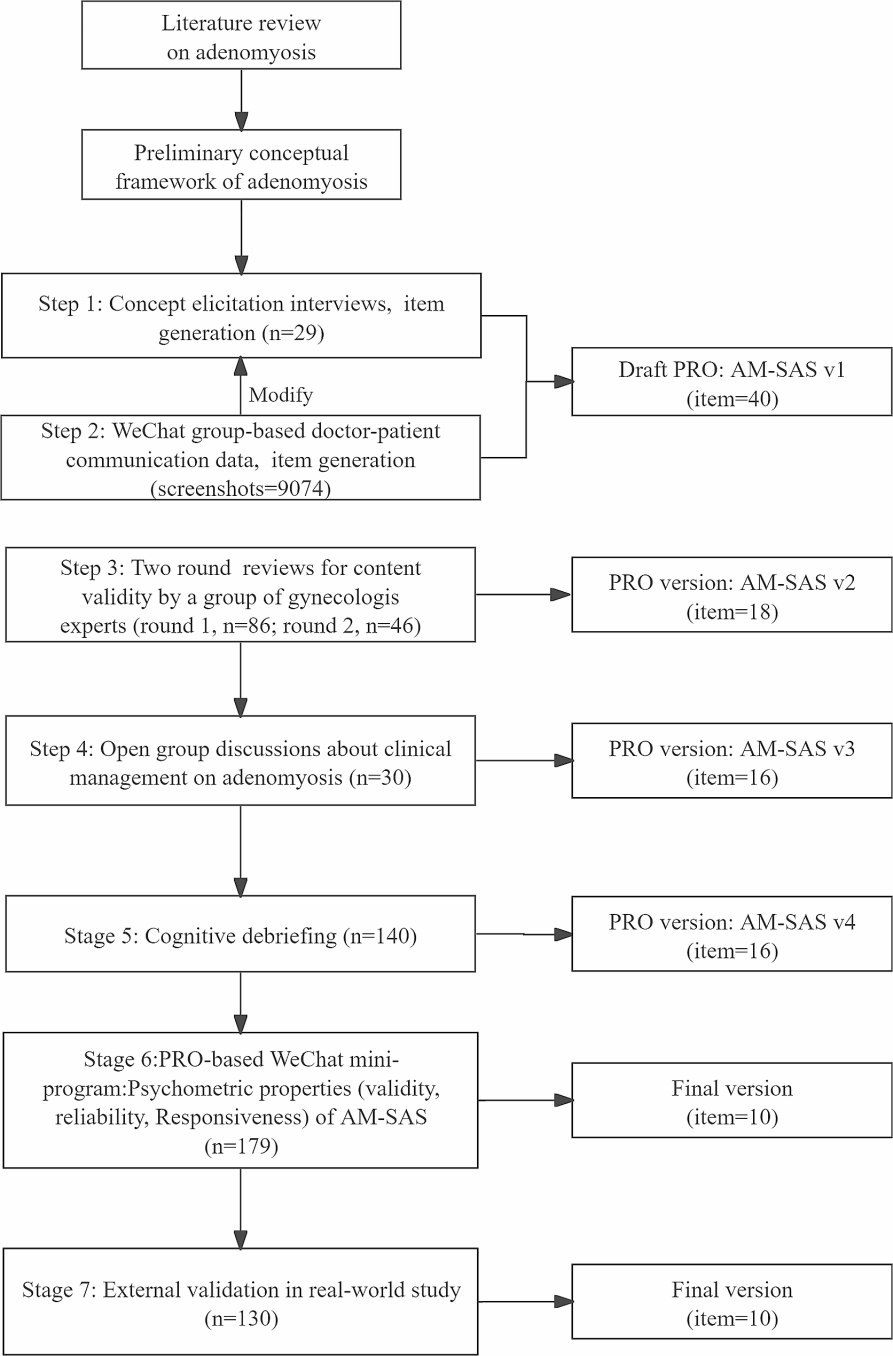
Flow chart of AM-SAS development and validation process. AM-SAS, core symptom management of adenomyosis

Three cohorts, screenshots of WeChat, and a cross-sectional study were used to develop, validate, and utilize the AM-SAS (Fig. 1). This trial was approved by the institutional review board of the Chongqing Medical University and three participating hospitals and registered in the Chinese Clinical Trial Registry (registration number ChiCTR2000038590). All patients provided written consent in accordance with the Declaration of Helsinki. Qualitative analysis was performed using the NVivo 12 (QSR International.2020. http://www.qsrinternational.com). Quantitative data analysis was conducted using JMP Clinical software version 6.1 (SAS Institute Inc., Cary, NC, USA). Correlations, means, standard deviations (SDs), ranges, 95% confidence limits, and frequencies were computed for all symptoms and subscales. Statistical significance was assumed at a 2-tailed a level of 0.05.

### Development of symptom monitoring instrument—AM-SAS

Data from Cohort 1 and screenshots of WeChat were used to generate potential symptom items from patient reports of symptom experiences. Cohort 1 comprised Chinese-speaking, non-menopausal patients 18 years of age or older with adenomyosis who received care at Nanchong Central Hospital between September and November of 2020. We exclude patients with fibroids, endometriosis or endometrial cancer [25]. Data from Cohort 2 was used to reduce the number of potential symptom items to those that were most relevant to patients. Cohort 2 was a panel of content experts comprising gynecologists from Nanchong Central Hospital, who have extensive experience caring for patients with adenomyosis.

#### Data collection methods and measures

In cohort 1, the patients participated in single, individual, face-to-face, in-depth, taped interviews about their symptom experiences since diagnosis. Trained qualitative research interviewers conducted all interviews, which were audiotaped and transcribed verbatim. The interview guide included open-ended questions about the patient’s symptoms of menstruation and non-menstruation, as well as the interference of symptoms with daily function, concerns, and anything else they wanted to share (See in supplement material). Additional probe questions were asked to elicit detailed information about the symptoms mentioned by the patient and to confirm that all important experiences were discussed. After the interview, patients provided sociodemographic information, previous treatment history for adenomyosis, and menstrual history. Laboratory information was collected from the patient’s medical record.

In screenshots of WeChat, we retrospectively collected free-text doctor-patient communication screenshots from WeChat in Haifu Hospital between January 1, 2019, to December 31, 2019. Haifu Hospital established the management process through the doctor-patient communication group. There were four management groups, comprising clinicians and nurse personnel who managed patients with adenomyosis.

In cohort 2, adenomyosis experts across China were surveyed on the WeChat (Tencent Corp) network using Sojump (Changsha ran Xing InfoTech Ltd) from December 4, 2020, to December 11, 2020. The survey contained an explanation of the study, a consent-to-participate statement, and an Expert Symptom Relevance (ESR) form. Three days after the first round of surveys, we began the second round with the same adenomyosis experts to guarantee consistency and stability of the items. The ESR form included symptoms and functional interference identified by Cohort 1. The relevance of each symptom to patients with adenomyosis was rated from 0 (not relevant) to 10 (very relevant). The experts could also suggest other relevant symptoms.

#### Data analysis

In cohort 1, the audiotaped patient interviews were transcribed professionally into text by a third-party transcription vendor. The transcripts were verified for accuracy by two researchers who cross-checked these with the content on the audiotapes. Using grounded theory methods [26], quotes were assigned a code determined by the underlying concept and grouped into higher level concepts [27]. Following analysis, researchers met to generate questionnaire items using a conceptual framework developed from the qualitative data. Clinical gynecologist experts were present to ensure the item concepts were accurate and relevant and that no clinically important symptoms or functional interference were missed (authors FX, YY, and XGZ). The researchers and clinicians jointly agreed on the final list of symptoms.

**Fig. 2:**
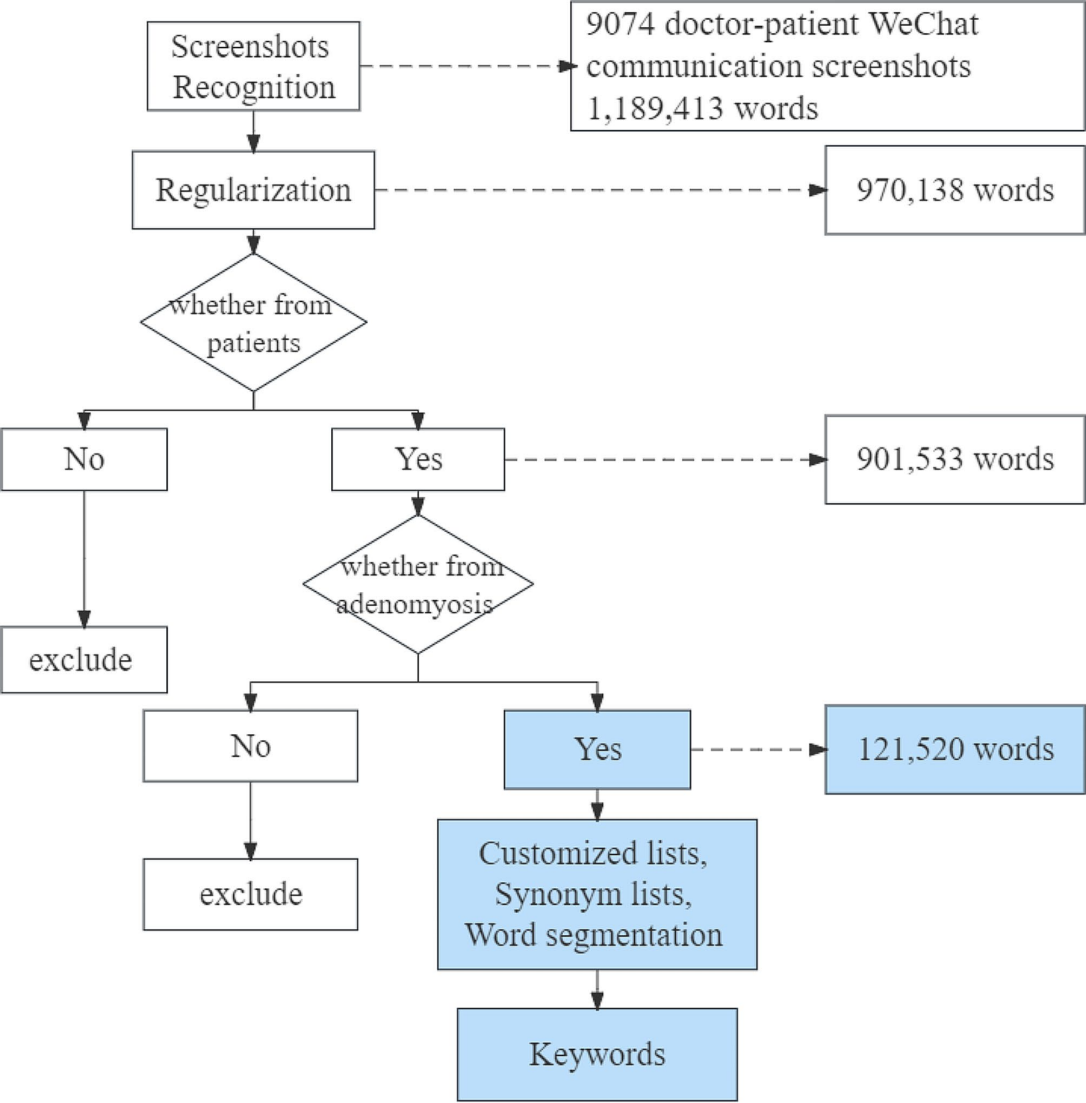
Natural language processing flow in doctor-patient communication screenshot

In screenshots of WeChat, after identifying screenshots as text, screening for symptoms reported by patients with adenomyosis, we established a custom dictionary, including a synonyms list, stop words, and realized automatic segment words. A flowchart of the process is shown in Fig. 2. Finally, we extracted symptom keywords with term frequency-inverse document frequency (TF-IDF). We compared the similarities and differences in extracted symptoms by different methods. Combining symptoms identified by the two methods, we generated a draft version of the symptom management scale for adenomyosis according to the expert discussion. Statistical analyses were conducted using Python via the Jupyter programming environment.

In cohort 2, the mean ratings of the individual items were calculated in the second survey round. All items that had a mean rating of > 4 in one of the two rounds of investigation or a difference rating of < 1 among the same experts were retained in a provisional AM-SAS for testing in Cohort 3.

### Validation of symptom monitoring instrument—AM-SAS

Cohort 3 reported symptoms regularly for 6 months, and the data was used to establish the reliability and validity of the final AM-SAS. Cohort 3 consisted of non-menopausal patients, 18 years of age or older, who were diagnosed with adenomyosis but did not receive a hysterectomy and who were being followed at Nanchong Central Hospital, The Affiliated Hospital of Southwest Medical University, or Haifu Hospital. All patients meeting the inclusion criteria from March to August 2021 were approached for study participation. We exclude patients with fibroid, endometriosis or endometrial cancer. Participated in the cross-sectional study, which was performed to externally validate the performance of the AM-SAS Symptoms domain. Adenomyosis was diagnosed by analyzing the Two-dimensional transvaginal ultrasound (TVUS) images of each patient by two sonographers with at least 5 years of work experience.

#### Data collection methods and measures

In cohort 3, all symptom measures data collect with WeChat mini-program. Each patient filled out the e-questionnaires of the AM-SAS, SF-36 through password-protected accounts on a personal electronic device; this was done once preoperatively (baseline) and 6 months following surgery.

This study included both inpatients (conservatively treated, excluding hysterectomy) and outpatients. The conservative treatment primarily consisted of High-Intensity Focused Ultrasound (HIFU) therapy. For baseline data, all inpatients filled out the AM-SAS and Short Form Health Survey (SF-36) on WeChat twice prior to surgery at an interval of > 24 h (for retest reliability). The AM-SAS was also collected 6 months following surgery. For outpatients, we collected the cross-sectional symptoms of patients with adenomyosis in AM-SAS. A clinical research coordinator collected demographic and clinical information from each patient’s medical record. The demographic questionnaire was that used for Cohort 1. Patients’ disease history and current clinical and treatment information were obtained as described for Cohort 1.

Patients participated in a cognitive-debriefing interview after completing the AM-SAS. The debriefing included ten detailed questions (i.e., covering item difficulty, comprehension, acceptability, preferred scoring, preferred data collection, etc.) to ensure the AM-SAS adequately measured the concepts and that items were correctly interpreted. Subquestions allowed patients to identify items that they found problematic. Further changes to symptom items could be made on the basis of the cognitive debriefing results.

Cross-sectional study included patients who received high-intensity focused ultrasound therapy for adenomyosis from Nanchong Central Hospital between 2017 and 2020. Patients were invited to complete the 10-item version of the AM-SAS to evaluate symptom burden during their most recent menstrual cycle.

#### Data analysis

In cohort 3, descriptive statistics were used to summarize the patient characteristics and demographics of Cohort 3. Item reduction was performed to identify the set of items that best represented the concept of interest [28]. Hierarchical cluster analysis was used to determine similar items among the AM-SAS specific items and their redundancy, by providing a graphical representation of how items cluster together in stages, unlike factor analysis where only the final clustering is shown. Clinicians were consulted before an item was removed to verify that it was not clinically relevant.

A combination of the Classical Test Theory (CTT) and Item Response Theory (IRT) was used to estimate the psychometric properties of the final AM-SAS. We used the Rating Scale Model of the Rasch measurement theory to evaluate item fit, reliability indices, item difficulty, uni-dimensionality, and construct validity.

The total Cronbach’s α and that with items deleted and the corrected item-total correlation coefficients were calculated to estimate the internal consistency reliability of each subscale of the AM-SAS. A Cronbach α value of 0.70 or higher indicates good internal consistent reliability [29]. Test-retest reliability was used to evaluate score reproducibility over time when the condition was stable, taken as “no change” on the patient-reported assessment of change item (24–72 h later). We calculated intraclass correlations for the AM-SAS subscales from Cohort 3 assessments made 2 consecutive days before conservative treatment to evaluate test-retest reliability. Content validity refers to the ability of an instrument to measure the concept of interest [18]. The content validity of the AM-SAS was established by patient input into item generation, selection, and confirmation by cognitive debriefing in the final validation stage. Concurrent validity refers to the correlation of an instrument with an instrument that measures a related but not identical concept [18]. Concurrent validity was examined with Spearman correlations of AM-SAS subscales scores and SF-36 subscales scores for the same patients. Known-group validity was defined as the ability of an instrument to identify outcomes of patients in specific groups using the instrument’s subscales or items when group differences are expected. We tested the AM-SAS sensitivity to different pain interference (not at all vs. yes) and anemia (yes vs. no) to establish known-group validity. Sensitivity was measured by Cohen’s D effect size. Sample size of patients was estimate as the 10 times of the number of items. We used cross-sectional study to verify the discriminant ability of AM-SAS in clinical application, AM-SAS was used to distinguish recurrent status.

## Results

### Development of symptom monitoring instrument—AM-SAS

#### Demographic and clinical characteristics of the study cohorts

The demographic and clinical characteristics of Cohorts 1 (*n* = 29) and 3 (*n* = 179) are summarized **in** Table 1. In Cohorts 1 and 3, the median age was 44 years, and approximately 65.5% (*n* = 19) and 48.6% (*n* = 87) of the patients received no prior treatment for adenomyosis, respectively. In Cohort 2, the first round (*n* = 86) comprised 79 (91.9%) gynecologists, 5 (5.8%) nurses, and 2 (2.9%) hospital administrators; 35 (40.7%) of the participants in Cohort 2 had over 15 years of work experience. The second round included 46 experts, 28 of whom responded twice. Demographic and clinical characteristics of Cohort 2 are shown in Table S1.

**Table 1:** Demographic and clinical characteristics of adenomyosis patients in study cohorts 1 and 3 at study entry

		Cohort 1: Concept Elicitation (*n* = 29)	Cohort 3: Psychometric validation (*n* = 179)	*P*
Age, y				0.95
	Mean ± SD	42.8 ± 5.7	42.6 ± 6.1	
	Median (IQR)	44 (37 to 47)	44 (39 to 47)	
Age at menarche (years)		13 (12 to 14)	13 (12 to 14)	0.32
BMI (kg/m^2^), mean ± SD		-	23.4 ± 3.7	-
Days menstrual bleeding, y		5 (4 to 7)	6 (5 to 7)	0.26
Days menstrual cycle, y		29 (28 to 30)	28 (25 to 30)	0.14
Clinic type				0.99^a^
	Outpatient	0(0%)	79(44.1%)	
	Hospitalization	29(100%)	100(55.9%)	
Education level				**0.03**
	Grade School or below	13(44.8%)	38(22.5%)	
	Middle School Graduate	8(27.6%)	63(37.3%)	
	High School Graduate or above	8(27.6%)	68(40.2%)	
Employment Status				0.13
	Unemployed	11(37.9%)	35(21.6%)	
	Employed Full-Time	9(31%)	67(41.4%)	
	other	9(31%)	60(37%)	
Gravidity				0.90
	0–2	6(21.4%)	49(28.8%)	
	3–4	17(60.7%)	75(44.1%)	
	5+	5(17.9%)	46(27.1%)	
Parity				0.35
	0	1(3.6%)	11(6.8%)	
	1	20(71.4%)	87(53.7%)	
	2+	7(25%)	64(39.5%)	
Abortions				0.71^a^
	0	3(10.3%)	20(12.4%)	
	1	4(13.8%)	36(22.4%)	
	2	10(34.5%)	44(27.3%)	
	3+	12(41.4%)	61(37.9%)	
Cesarean section				**0.008**
	No	25(86.2%)	84(60.4%)	
	Yes	4(13.8%)	55(39.6%)	
Adenomyosis				
	No	19(65.5%)	87(48.6%)	
	Yes	10(34.5%)	92(51.4%)	
Prior treatment of Adenomyosis (Multi-choice)				0.24^a^
	Untreated	19(65.5%)	87 (48.3%)	
	High Intensity Focused Ultrasound	0(0%)	21 (11.7%)	
	Medicine (include GnRH)	2(6.9%)	52 (28.9%)	
	Mirena	4(13.8%)	24 (13.3%)	
	Diagnostic curettage	4(13.8%)	14 (7.8%)	
	Other (e.g. moxa-moxibustion, laparoscope, etc.)	0(0%)	10 (5.6%)	
Anemia				**0.002**
	No	14(48.3%)	56(31.3%)	
	Yes	13(44.8%)	49(27.4%)	
	Unknown	2(6.9%)	74(41.3%)	
Uterine volume (cm^3^)		-	191.8 ± 114.9	-

^a^ Using 2-tailed Fisher exact test for categorical variables

The values with statistical significance present as bold type

#### Generation of potential symptom items from patient reports: cohort 1 and WeChat screenshots

**Fig. 3:**
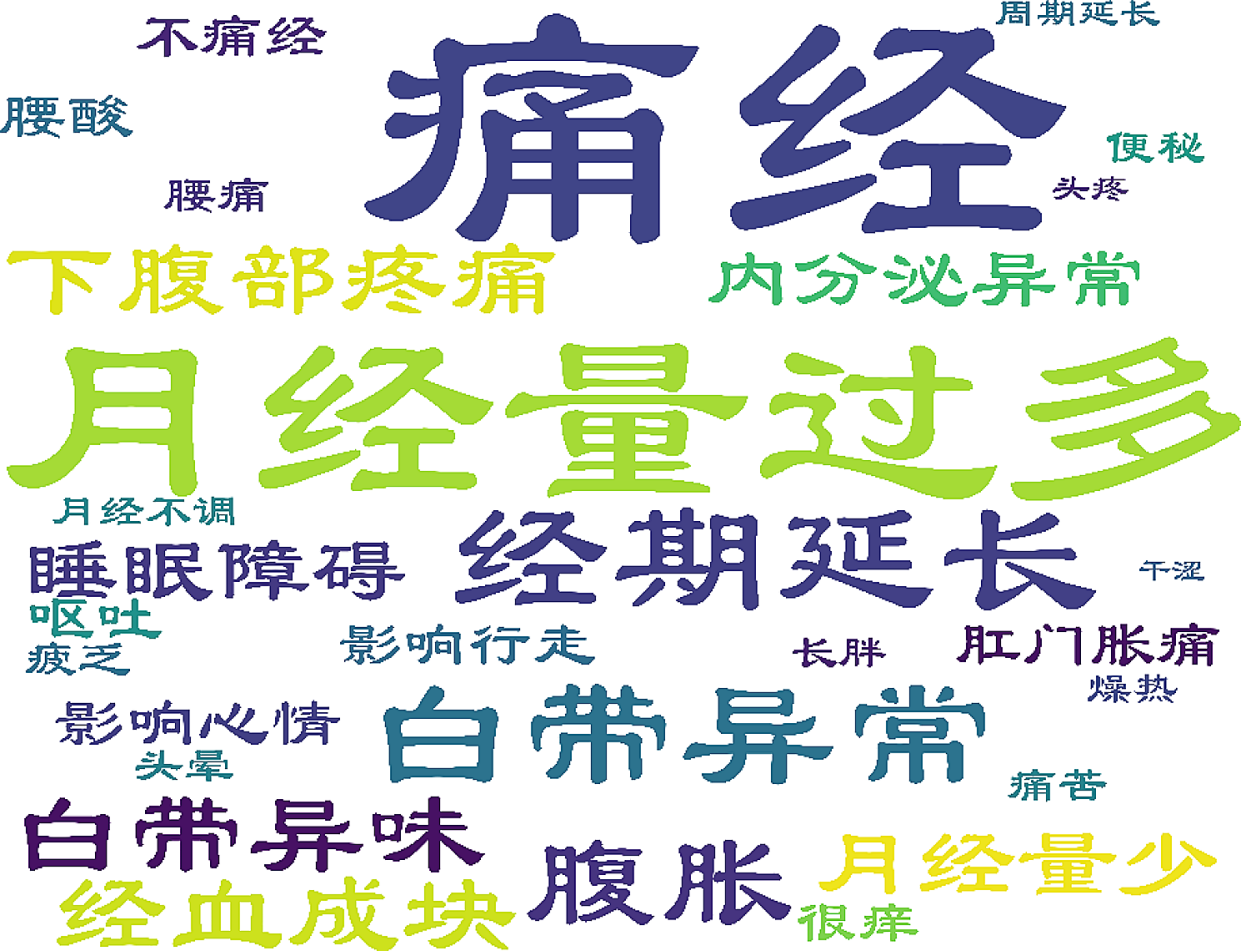
Symptoms word cloud extracted by TF-IDF

Twenty-eight symptoms occurring over the disease course were identified from content analysis of the 29 qualitative interviews; of these, 18 were reported by at least 20% of patients. Candidates were generated using terms consistent with words used by patients in the interviews. A total of 9,074 doctor-patient communication screenshots were identified on WeChat, comprising 1,189,413 words. NLP extracted 121,520 words from the patient reports, while TF-IDF identified 29 symptoms (Fig. 3). We summarized the symptoms identified from the two methods.

Two clinicians and two researchers reviewed the symptom item list. Although the symptoms of swollen breasts and leg pain were reported by less than 20% of the patients, clinicians still suggested including them as symptom items. Expert consideration of the extracted symptoms from WeChat suggested deleting hypomenorrhea, no dysmenorrhea, vaginal itching, increase in body weight, vaginal dryness, etc. We also excluded dyspareunia, but kept “sexual life interference.” Table 2 depicts the extracted symptoms from the two methods. Finally, a candidate list of 18 symptoms (AM-SAS v1) was developed by consensus of the researchers and clinicians for rating by the expert panel.

**Table 2:** Comparison the symptoms extracted from WeChat and qualitative interview

Draft version V1	Common symptoms	WeChat specific symptoms	Qualitative interview specific symptoms
Menorrhagia	Menorrhagia	Headache	Leg pain
Abdominal pain	Abdominal pain	Hypomenorrhea^a^	Swollen breasts
Waist pain (soreness)	Waist pain (soreness)	Passing blood clots^a^	Frequent urination
Abnormal bearing-down pain	Abnormal bearing-down pain	No dysmenorrhea^a^	Lack of appetite
Anus bearing-down pain	Anus bearing-down pain	Vaginal itching^a^	Work interference
Dizzy	Dizzy	Increase in body weight ^a^	Social contact interference
Fatigue	Fatigue	Vaginal dryness^a^	Sexual life interference
Disturbed Sleep	Disturbed Sleep	Hot flush^a^	Dry mouth (< 20%)
Vomiting	Vomiting		Cold sweat (< 20%)
Mood interference	Mood interference		Flustered (< 20%)
Walking interference	Walking interference		Pelvic pain (< 20%)
Frequent urination/ Constipation	Constipation		Chest pain (< 20%)
Headache	Leucorrhea abnormality (< 20%)		Nausea (< 20%)
Leg pain	Leucorrhoea peculiar smell (< 20%)		Diarrhea (< 20%)
Swollen breasts	Prolonged menstruation^b^		Sexual intercourse pain (keep Sexual life interference)
Lack of appetite	Menstrual extension^b^		
Work interference	Dysmenorrhea^c^		
Social contact interference			
Sexual life interference			

^a^ Not a specific symptom of adenomyosis

^b^ Not subjective symptom

^c^ Refine into several pain

#### Reduction of symptom items by expert panel rating: cohort 2

Because leg pain and headache rated < 4 in the two-round expert panel, and the difference rating for swollen breasts was > 1, according to the criteria for inclusion, we deleted those symptoms from the scale (Table S2). Although not spontaneously mentioned during the expert panel rating, menorrhagia was reported by 20 experts; thus, it was added to the AM-SAS. Finally, 16 items in the AM-SAS underwent further psychometric validation.

### Validation of symptom monitoring instrument—AM-SAS

#### Cognitive debriefing

The participants rated the provisional AM-SAS questions as easy to understand and complete, with a mean (SD) difficulty score of 1.62 (2.38) (median [interquartile range] of 0 [0 to 3]). Among the total patients, 117 (80.7%) reported the 0 to 10 scoring system as easy to understand and use and were comfortable grading symptoms, and 122 (84.1%) were willing to continue to fill-out the AM-SAS.

#### Description of AM-SAS scores and item reduction

Means and SDs for the individual symptom and interference items and all subscales for both the test and retest scores are shown in Table S3.

The five symptoms with the highest mean severity were abdominal pain, menorrhagia, abnormal bearing-down pain, waist pain, and fatigue. The five symptoms with the highest moderate-to-severe (rating ≥ 4) incidence were abdominal cramping (69.27%), menorrhagia (51.96%), abnormal bearing-down pain (49.16%), waist pain (47.49%), and fatigue (46.37%).

We used hierarchical cluster analysis to identify groups of similar items (Figure S1). Based on the cluster analysis and clinical judgment considering our data on symptom prevalence and severity, we combined abnormal bearing-down pain and abdominal pain into abdominal cramping. Besides, four of the initial 16 items were eliminated: frequent urination, dizzy, difficulty in walking, and social interaction. Mood and work were highly related based on the cluster analysis. This analysis suggested that one of these items could be eliminated. However, clinicians argued that mood and work represent the physical and psychological disturbances, respectively, and that both items should be included in the AM-SAS list. At last, gynecologist found that nausea/vomiting showed low symptom burden (with means of 1.1 and 2.1, respectively), and they were subsequently deleted from the list.

The final list included 10 items: menorrhagia, abdominal cramping, waist pain, anus bearing-down pain, fatigue, disturbed sleep, lack of appetite, mood, work, and sexual life interference.

#### Rasch validation

To portray the overall measurement properties of the AM-SAS scale, the summary statistics and the item-person variable map were first examined. As shown in Table S4, the mean measure for persons was − 0.3 logits, whereas that for items was set by default at 0.00, indicating that the whole person ability was approximately equal to the item difficulty. Table S4 indicates that the separation index for persons was 1.91, and that for items was 4.85, suggesting that there were at least two distinguishable strata of patients with a latent trait being assessed by the AM-SAS scale, and about five distinguishable levels of item difficulty. The reliability for persons and items was 0.81 and 0.96, respectively, both of which were above the acceptable threshold of 0.8 [30]. The summary statistics yielded by Rasch analysis seems to indicate the overall satisfactory measurement properties of the AM-SAS scale.

The results of Rasch item analysis are presented in Table S5. The second column of this table shows the item difficulties calibrated by the Rasch analysis. As indicated by the statistics in this column, the items were calibrated at varying difficulty levels, ranging from − 0.35 to 0.22 logits. An examination of the point measure (PTMEA) correlations in this table indicated that all items exhibited moderate to strong correlations (0.57–0.66), suggesting that these items functioned in the same direction and were closely related to the latent trait. The high PTMEA correlations also lent support to the uni-dimensionality of the AM-SAS scale, which is an important principle of the Rasch analysis [30]. The uni-dimensionality of the AM-SAS was further verified by the observation that 51.5% of the variance was explained by the Rasch measure, which was above the criterion of 50% suggested by Linacre [30]. Given that the purpose of the current study was to examine the validity of an AM-SAS used for low-stakes placement decisions, the in- and outfit MnSq range of 0.6 to 1.4 was adopted [31]. As the statistics in Table S5 show, excluding two items (i.e., menorrhagia and sexual life interference), all other items fit in this range (i.e., 0.6–1.4), suggesting that the items in the AM-SAS scale fit the Rasch model sufficiently well to define a common construct, i.e., patients’ self-assessment of their symptom severity.

#### CTT validation

The AM-SAS symptom/interference scales showed good internal consistency reliability (Table 3). The data support a high internal consistency with a Cronbach’s coefficient alpha of 0.85 (10 symptoms’ severity), and a total Cronbach’s α of > 0.8 for each item. The intraclass correlations of the AM-SAS total symptom and interference scales and subscales administered after 24 h (*n* = 51) indicated good test-retest reliability. All values were > 0.7, except mood and work (Table 3).

**Table 3:** Internal consistency and test-retest reliability of the AM-SAS

Scale		Cronbach coefficient α internal consistency				Test-retest^a^		
		Test sample Cronbach’s α (*n* = 179)	Total cronbach’s α if item deleted	Retest sample Cronbach’s α (*n* = 51)	Total cronbach’s α if item deleted	Intraclass correlation	*P*	Mean (SD) in test sample
All symptom severity		0.85		0.89				
	Menorrhagia		0.853		0.897	0.710	< 0.001	4.1(3.4)
	Abdominal cramping		0.830		0.88	0.836	< 0.001	5.5(3.0)
	Waist pain		0.826		0.878	0.780	< 0.001	3.9(3.1)
	Anus bearing-down pain		0.836		0.881	0.788	< 0.001	2.3(2.6)
	Fatigue		0.818		0.876	0.711	< 0.001	3.9(3.0)
	Disturbed Sleep		0.827		0.883	0.828	< 0.001	3.4(3.2)
	Lack of appetite		0.826		0.885	0.706	< 0.001	2.2(2.9)
	Mood interference		0.817		0.877	0.697	< 0.001	3.6(3.1)
	Work interference		0.828		0.882	0.650	< 0.001	3.6(3.5)
	Sexual life interference		0.845		0.883	0.707	< 0.001	2.3(2.9)

^a^The time interval between test and retest was 24 h before conservative treatment

Results of cognitive debriefing provided evidence of content validity. Concurrent validity was established by correlating the mean total symptom scores from the AM-SAS with those of the SF-36 subscales for the same patients. Spearman correlations were between − 0.31 and − 0.48 and were statistically significant, except for Role-Physical. Symptom severity was moderately correlated to the overall SF-36. A Spearman product moment correlation showed that menorrhagia was moderately correlated with the anemia status (*r* = 0.37, *P* < 0.001) and self-reported menorrhagia (*r* = 0.31, *P* < 0.001).

We used *t* tests to explore the differences in the mean symptom severity by pain interference with normal work (not at all vs. yes) (Table 4). Patients who reported interference with work reported significantly increased symptom severity (3.31, SD = 2.00 vs. 1.74, SD = 1.36; *P* < 0.001), with an effect size (Cohen’s d) of 0.8. To evaluate the sensitivity of menorrhagia in discriminating anemia severity, patients were divided into two groups based on hemoglobin (HGB) values. Those patients with an HGB value of 110 pg/mL or less were categorized as anemia (*n* = 46, 47.9%) and those patients with a HGB value of greater than 110 to 150 pg/mL were categorized as normal (*n* = 50, 52.1%). As predicted, there was a significant difference in the severity of menorrhagia between the anemic and normal HGB groups (3.04, SD = 3.17 vs. 5.68, SD = 3.41; *P* < 0.001), with an effect size (Cohen’s d) of 0.66.

**Table 4:** Comparison of symptom severity in AM-SAS by anemia severity and pain interfere

Menorrhagia		Pain interfere with your normal work				
		Normal (*n* = 50)	Anemia (*n* = 46)	Mean difference	Effect size	*P*
		3.04 ± 3.17	5.68 ± 3.41	-2.63 (-3.97 to -3.97)	-0.66	**< 0.001**
		Pain interfere with your normal work				
		Not at all (*n* = 27)	Yes (*n* = 101)	Mean difference	Effect size	*P*
Symptom severity						
	Abdominal cramping	4.74 ± 3.19	5.5 ± 3.07	-0.8(-2.1 to 0.6)	-0.26	0.26
	Waist pain	3.22 ± 2.34	3.99 ± 3.27	-0.8(-2.1 to 0.6)	-0.24	0.26
	Anus bearing-down pain	1.33 ± 1.86	2.43 ± 2.84	-1.1(-2.2 to 0)	-0.39	**0.02**
	Fatigue	2.7 ± 2.67	4.07 ± 3.2	-1.4(-2.7 to 0)	-0.44	**0.04**
	Disturbed Sleep	1.63 ± 2.73	4 ± 3.25	-2.4(-3.7 to -1)	-0.74	**< 0.001**
	Lack of appetite	0.63 ± 1.57	2.28 ± 3.01	-1.7(-2.8 to -0.5)	-0.56	**< 0.001**
	Mood interference	1.85 ± 2.84	3.87 ± 3.14	-2(-3.3 to -0.7)	-0.64	**0.003**
	Work interference	1.6 ± 3.04	3.81 ± 3.51	-2.2(-3.7 to -0.7)	-0.63	**0.005**
	Sexual life interference	0.63 ± 1.21	2.32 ± 2.87	-1.7(-2.8 to -0.6)	-0.59	**< 0.001**

The values with statistical significance present as **bold type**

#### External validation AM-SAS in a real-word study

In cross-sectional study, 130 post-treatment patients with well-controlled disease and stable symptom severity met the eligibility criteria for analysis and completed the AM-SAS measure; the mean symptom severity was less than 3 points for each item (Table S6). The recurrence rate was 22.6%. We used the AM-SAS to distinguish the symptom severity between relapse and no-relapse patients. Compared with patients without recurrence, those who did report relapse showed higher symptom burden and poorer physical status (Table 5). The Engelish version of AM-SAS showed in Table S7.

**Table 5:** Using AM-SAS to examine difference in symptom and functional burden between none-relapse and relapse with adenomyosis

Scale		Recurrence (*n* = 28) ^a^	Without recurrence (*n* = 54) ^a^	*P*
Means of symptom severity		1.71 (1.29 to 2.71)	0.71 (0.29 to 1.43)	**< 0.001**
	Menorrhagia	2 (0.5 to 5)	0 (0 to 1)	**0.001**
	Abdominal cramping	4 (2 to 5.5)	1.5 (0 to 2)	**< 0.001**
	Waist pain	2 (0.5 to 4)	1 (0 to 2)	**0.025**
	Anus bearing-down pain	0 (0 to 2)	0 (0 to 0)	**0.012**
	Fatigue	0.5 (0 to 2)	0 (0 to 1)	0.260
	Disturbed Sleep	2 (0 to 3)	0 (0 to 2)	**0.015**
	Lack of appetite	0 (0 to 0)	0 (0 to 0)	0.194
Means of function interference		0.33 (0 to 1.67)	0 (0 to 0.67)	**0.028**
	Mood interference	0.5 (0 to 3)	0 (0 to 1)	0.058
	Work interference	0 (0 to 2)	0 (0 to 0)	**0.015**
	Sexual life interference	0 (0 to 0)	0 (0 to 0)	0.215

^a^ Median (P25 to P75)

The values with statistical significance present as bold type

#### Defined index symptom for routine management

In Cohort 3, the mean symptom score of the 10 items showed that menorrhagia (4.1 ± 3.4) and abdominal cramping (5.4 ± 3.0) were moderate-to-severe. Moreover, 69.3% patients reported moderate to-severe (rating ≥ 4) abdominal cramping, and 51.2% patients reported menorrhagia. Rasch analysis showed that 40.5% of the variance was explained if only menorrhagia and abdominal cramping were used, totaling 51.5% of the 10 items. Furthermore, 143 (80.0%) patients reported either moderate-to-severe menorrhagia or abdominal cramping. In Cohort 4, 28 patients reported adenomyosis recurrence, 25 (89.3%) of whom reported either menorrhagia or abdominal cramping. These results suggested that abdominal cramping and menorrhagia may be used as an index symptom for follow-up and routine management.

## Discussion

Based on Wechat clinician-patient group communication data, we first constructed symptom corpus and the method of extracting keywords in free text containing a large amount of noise. The AM-SAS was developed with peer review to obtain clinician input, qualitative interviews and Wechat clinician-patient group communication for obtaining patient input. It consists of 10 items of adenomyosis-specific symptoms were retained for the final instrument and were validated psychometrically. Our results provide psychometric evidence for the use of the AM-SAS, which is the first broadly applicable, adenomyosis-specific PRO tool to be developed with extensive patient involvement and the first to evaluate the pre- and postoperative symptom burden. As an outcome measure of multiple symptoms in clinical trials, the AM-SAS may identify patients who need extensive care after discharge and capture significant beneficial changes of patients may have been overlooked.

The WeChat platform has previously been utilized in individuals with HIV infection, those undergoing a dementia-specific training program, and those requiring bowel preparation for outpatient colonoscopy, whereupon it provided an easy, economical, efficient, and convenient intervention mode for clinical staff [21]. Compared to a qualitative interview, WeChat group-based doctor-patient communication texts identified by NLP can catch most symptoms reported by patients to supplement the symptom lists for interview. However, items of privacy were missed due to the privacy of the mute-person communication group; for example, dyspareunia and breast tenderness, were not mentioned in the WeChat group-based data. At the same time, surgery-related symptoms were extracted from WeChat, including hypomenorrhea, vaginal itching, and vaginal dryness, which are symptoms of postoperative recovery or postoperative vaginitis. To preserve the integrity and accuracy of the symptoms, and to avoid irrelevant symptoms in the final version, we used expert consultation after NLP. We will further determine whether NLP extracted symptom items based on the WeChat group can replace part of the expert interviews and manual identification for PRO instrument development in the future.

Small proportions of missing data and the brevity of 10-item assessment point to the acceptability of the scale for monitoring and managing patients, and the availability of real-time monitoring and the ease of use reported by patients supports the module’s minimum burden on patients in the target population. Validated by Rasch and the CTT, the AM-SAS has high internal consistency reliability, and the measure has good concurrent, convergent, and discriminant validity. In the development and external validation cohort, we provide evidence that the AM-SAS can discriminate among patients with differing levels of pain-interference, and among those who experience anemia and recurrence. Further testing of the tool’s ability to discriminate patients with adenomyosis from those with other similar conditions (e.g., endometriosis, chocolate cyst) would be a valuable avenue for future research.

The AM-SAS can be used in both clinical practice and clinical trials to evaluate patient’s symptom burden during perioperative care, even with frequent assessments [31, 32]. This study provides useful and precise information about the relevant symptom burden and interference caused by adenomyosis. We found that, in outpatients and inpatients, over **60%** of patients reported moderate to-severe (rating ≥ 4) abdominal cramping, and over **50%** of patients reported menorrhagia. As expected, abdominal cramping and menorrhagia were the most severe symptom across the pre- and post-treatment period. Long-term and multi-frequent symptom management of adenomyosis is always challenging because of its high rate of recurrence, requiring intensive monitoring after discharge [33]. as screening index symptoms in long-term care to improve the efficiency and reduce the burden of patients and medical staff on management.

According to the high symptom burden of menorrhagia and abdominal cramping, and their high correlation with recurrence we proposed them as index symptoms for long-term, effective extensive care to monitor recurrence after therapy. Once either of the screening symptoms exceeded a threshold value, patients reported specific symptom items to evaluate the recurrence status. The screening process could enable a comprehensive assessment of symptom status and timely clinical intervention for patients with a high risk of recurrence. In addition, the screening process could improve the efficiency of management, reducing the burden of patients and medical staff. Further, these symptoms could be targeted for intensive monitoring in routine patient care to obtain evidence on the effectiveness of drugs and treatments. We will further validate the screening accuracy and practicability of index symptoms in a future real-world study.

Although several studies indicated electronic data collection as a convenient way of implementing a continuous follow-up in regular time [34, 35], especially in electronic devices for home monitoring [36, 37], the follow-up timepoint is dynamic and unbalanced in patients with adenomyosis and menstrual disorders, especially after treatment, such as gonadotropin-releasing hormone (GnRH) cessation of menstruation to-be-decided [33]. To pinpoint the date of each menstrual period to monitor the patient’s symptom burden, a flexible schedule must be developed in advance, especially to establish the trigger event so the platform can send follow-up information to patients, which poses a challenge to the available technology of the follow-up system. In this study, we set electronic monitoring platforms multiple rounds of sending reminders, which probably reduced the response rate and patient’s compliance.

Therefore, we combined conventional (telephone follow-up) and electronic surveillance methods to ensure timeliness and a higher participation rate. Although electronic monitoring is widely used, there are obstacles to its use in real-world applications [38], such as potential delays [39] and low response rates; thus traditional manual follow-up still plays an important role in management. The key to resolving these limitations is to consider how to change the passive follow-up pattern to one in which patients actively report symptoms, which means patients actively report to the electronic monitoring system when menstruating. The key to resolving these limitations is to consider applications of artificial intelligence (AI) in the management of adenomyosis. AI can play a pivotal role in enhancing patient compliance and managing the dynamic nature of follow-up care for adenomyosis and menstrual disorders. By leveraging AI-driven predictive analytics, the timing of menstrual periods can be estimated with greater accuracy, allowing for the development of adaptive reminder systems that are more aligned with the patient’s individual cycle [40]. This proactive approach can lead to more personalized and timely interventions, potentially improving patient outcomes and satisfaction. Therefore we can change the passive follow-up pattern to one in which patients actively report symptoms, which means patients actively report to the electronic monitoring system when menstruating.

As a strength of this study, we referenced the development process of newly created PRO instruments from the FDA to develop and validate the AM-SAS to design a well-defined and reliable PRO tool to guarantee accurate and sensitive symptom assessment. Innovatively, we used NLP to automatically extract patient-reported symptoms in WeChat to supplement deficiency-specific symptoms in AM-SAS. We used external validation to verify the applicability of the scale. We also propose an index management symptom for clinical practice to improve patient quality of care and reduce the burden of patients and medical staff on management. We suggest menorrhagia and abdominal cramping as screening symptoms for long-term and effective PRO monitoring recurrence after therapy.

Our study had limitations. First, we had access to a large population of patients with adenomyosis but only completed accrual within 6 months; thus, there is a lack of long-term description of symptom burden after treatment. However, we plan to continue to monitor patient symptoms consistently over 1 year. Second, the external cohort was a homogenous group of patients after surgery with well-controlled disease and stable symptom severity, which was below the symptom status quo of most patients with adenomyosis. However, we used cross-sectional data to psychometrically validate the results. Third, the two items proposed as screening symptoms require further clinical study.

## Conclusion

Our study demonstrated that Electronic PRO-based AM-SAS based on WeChat mini-program is a useful instrument for tracking specific symptoms in patients undergoing management for adenomyosis. WeChat group-based doctor-patient communication texts identified by NLP can catch most symptoms reported by patients to supplement the symptom lists for interview. A significant proportion of patients with adenomyosis experience moderate-to-severe symptoms. These patients require attention to manage symptoms and maintain functioning. As an outcome measure of multiple symptoms in clinical trials, the AM-SAS may identify patients who need extensive care after discharge and capture significant beneficial changes of patients may have been overlooked. With AM-SAS practical application, may improve patient quality of care and promote the timely commencement of reminders and treatment if symptom recurrence.

### Electronic supplementary material

Below is the link to the electronic supplementary material.

Supplementary Material 1

Supplementary Material 2

## Data Availability

All data in the main text and the Supplementary Information have been uploaded into a website (https://cdo.epro-vision.com:81/eproPad/html/index.html). Readers have read access to the data but are not allowed to export the data. Full access to the data will be available to researchers for the purposes of research or regulatory decision-making with a signed data access agreement after approval of a proposal. All data requests will be reviewed by the research committee at Chongqing Medical University and the corresponding authors to verify whether the request is subject to any intellectual property or confidentiality obligations.

## Declarations

### Disclaimer

As submitting author, I attest that I have written consent from all authors to submit the article, and that all authors accept complete responsibility for the contents of this article. This article has not been previously published. Finally, I attest that the article is independent and original work, and all conclusions are from the authors.
