# Supplementary material for: WeChat assisted electronic symptom measurement for patients with adenomyosis
Source: BMC Med Inform Decis Mak. 2024 Jun 17;24:168. doi: 10.1186/s12911-024-02570-8 (PMC11181603; doi:10.1186/s12911-024-02570-8)
Supplement: Supplementary file 1 — Supplementary Material 1 [file 12911_2024_2570_MOESM1_ESM.docx]

**Novel Technical Contribution:**

- Development of the AM-SAS: The creation of a new, patient-reported outcome (PRO) instrument specifically for adenomyosis, which incorporates patient experiences and clinician insights to assess disease symptoms comprehensively.
- Integration of WeChat Mini-Program: The innovative use of a WeChat mini-program for real-time symptom monitoring, which leverages the widespread use of WeChat in China for efficient patient engagement and data collection.
- Application of Natural Language Processing (NLP): The employment of NLP to extract and analyze data from unstructured WeChat communications, providing a more nuanced understanding of patient-reported symptoms and supplementing the qualitative interview data.

**Strengths:**

- Patient-Centric Approach: The AM-SAS was developed with extensive patient involvement, ensuring that the instrument is relevant and responsive to the needs and experiences of individuals with adenomyosis.
- Real-Time Monitoring: The use of a WeChat mini-program allows for timely and continuous monitoring of symptoms, which can lead to more immediate and effective clinical interventions.
- Data Collection Efficiency: The combination of NLP with traditional interviews enhances the efficiency of data collection, reducing the potential for bias and increasing the accuracy of symptom identification.

**Weaknesses:**

- Limited Long-Term Data: The study's collection of data over a relatively short period may not fully capture the long-term symptomatology and treatment outcomes for adenomyosis.
- Technical Challenges: The reliance on electronic monitoring systems and the need for a flexible schedule to track menstrual periods present technical challenges, including potential delays and low response rates, which may affect data accuracy and patient compliance.
