# Supplementary material for: WeChat assisted electronic symptom measurement for patients with adenomyosis
Source: BMC Med Inform Decis Mak. 2024 Jun 17;24:168. doi: 10.1186/s12911-024-02570-8 (PMC11181603; doi:10.1186/s12911-024-02570-8)
Supplement: Supplementary file 2 — Supplementary Material 2 [file 12911_2024_2570_MOESM2_ESM.docx]

**Supplemental Digital Content**

**WeChat assisted Electronic symptom measurement for Patients with Adenomyosis**

**Wei Xu^1^, Xin Zhang^2^, Fan Xu^3^, Yuan Yuan^4^, Ying Tang^3^****, Qiuling Shi*, PhD* ^1,2,✉^**

*^1^* College of Public Health, Chongqing Medical University, Chongqing, China

^2^ State Key Laboratory of Ultrasound in Medicine and Engineering, College of Biomedical Engineering, Chongqing Medical University, Chongqing, China

^3^ Department of Obstetrics and Gynecology, Nanchong Central Hospital, Second Clinical Medical College, North Sichuan Medical University, Nanchong, Sichuan, China

^4^ Department of Obstetrics and Gynecology, Affiliated Hospital of Southwest Medical University, Luzhou, Sichuan, China

**^✉^ Address for correspondence:**

Professor Qiuling Shi

College of Public Health, Chongqing Medical University, Chongqing, China

No. 1 Yixueyuan Road, Yuzhong District, Chongqing 400016, P.R.China

Tel: +86 182 9058 5397

E-mail address: [qshi@cqmu.edu.cn](mailto:qshi@cqmu.edu.cn) (Qiuling Shi)

**Patient Interview Open-Ended Questions**

1.Please describe all the symptoms you experience during your menstrual period.

2.Please further elaborate on the following:

a. What are the main symptoms of your dysmenorrhea during menstruation? Where does it hurt?. How long do these symptoms last?

b. Which of the above symptoms do you consider the most severe? Why?

c. Do you experience pain only during menstruation or also outside of menstruation? d. Do these symptoms affect your activities? If so, which ones?

e. Which restricted activity bothers you the most after menstruation?

f. Is there anything else you would like to tell us about your symptoms or experiences?


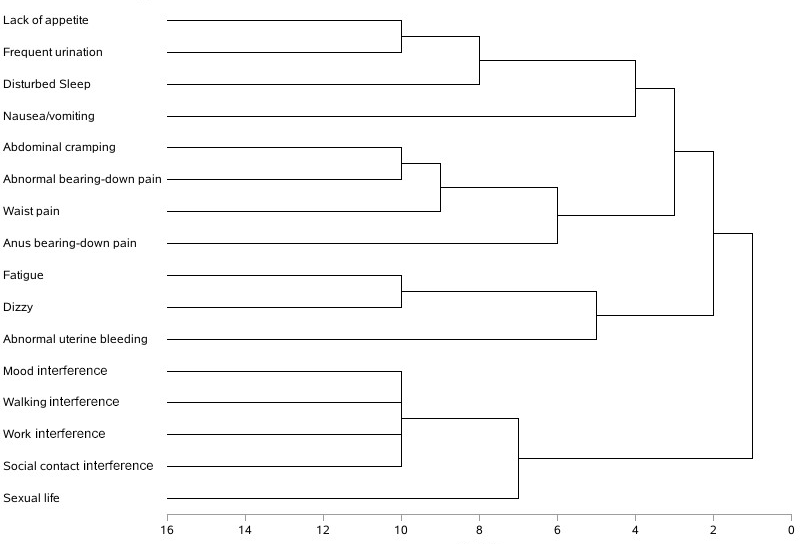


**Figure S1**. Hierarchical cluster analysis for the AM-SAS.

**Table S1.** Demographic characteristics of experts at wide experience caring for patients with adenomyosis in study cohorts 2

|  |  | Round 1  (n=86) | Round 1 and 2 (n=28) |
| --- | --- | --- | --- |
| Gender |  |  |  |
|  | Male | 13(15.1%) | 6(21.4%) |
|  | Female | 73(84.9%) | 22(78.6%) |
| Age |  |  |  |
|  | 20-29 | 12(14%) | 3(10.7%) |
|  | 30-39 | 42(48.8%) | 17(60.7%) |
|  | 40-49 | 24(27.9%) | 7(25%) |
|  | older than 50 | 8(9.3%) | 1(3.6%) |
| Occupation | |  |  |
|  | Gynecologist | 79(91.9%) | 26(92.9%) |
|  | Nurses | 5(5.8%) |  |
|  | Others  (Hospital administrators, researcher) | 2(2.3%) | 2(7.1%) |
| Education level | |  |  |
|  | Associate | 4(4.7%) | 1(3.6%) |
|  | Bachelor | 34(39.5%) | 10(35.7%) |
|  | Master | 33(38.4%) | 10(35.7%) |
|  | Doctor | 15(17.4%) | 7(25%) |
| Professional title | |  |  |
|  | Primary title | 15(17.4%) | 2(7.1%) |
|  | Middle title | 35(40.7%) | 15(53.6%) |
|  | High title | 36(41.9%) | 11(39.3%) |
| Years of work experience | |  |  |
|  | 1-5 | 14(16.3%) | 4(14.3%) |
|  | 6-10 | 21(24.4%) | 8(28.6%) |
|  | 11-15 | 16(18.6%) | 6(21.4%) |
|  | >15 | 35(40.7%) | 10(35.7%) |

**Table S2**. Symptom items scoring by expert panel rating in two rounds. The deleted symptom presents as bold type.

|  | Round 1 (n=86) | | | Round 2 (n=46) | | |  | Round 1 and 2 (n=28) | | |
| --- | --- | --- | --- | --- | --- | --- | --- | --- | --- | --- |
| Items | Range | Mean | SD | Range | Mean | SD | Items | Range | difference means | SD |
| Swollen breasts | 0 to 9 | 3.13 | 2.29 | 0 to 8 | 4.26 | 2.65 | Lack of appetite | -5 to 4 | 0.50 | 1.86 |
| **Leg pain** | 0 to 9 | **3.35** | 2.52 | 0 to 9 | **3.63** | 2.09 | Leg pain | -5 to 3 | -0.50 | 1.50 |
| **Headache** | 0 to 10 | **3.41** | 2.50 | 0 to 10 | **3.85** | 2.17 | Abnormal bearing-down pain | -3 to 2 | -0.11 | 1.26 |
| Dizzy | 0 to 10 | 3.64 | 2.80 | 0 to 10 | 4.33 | 2.68 | Social contact interference | -4 to 4 | -0.14 | 1.80 |
| Frequent urination | 0 to 10 | 3.97 | 2.50 | 0 to 10 | 4.46 | 2.49 | Anus bearing-down pain | -3 to 2 | -0.18 | 1.12 |
| Lack of appetite | 0 to 10 | 4.05 | 2.52 | 0 to 10 | 4.24 | 2.63 | Dizzy | -4 to 4 | -0.18 | 2.09 |
| Fatigue | 0 to 10 | 4.36 | 2.62 | 0 to 8 | 5.02 | 2.61 | Waist pain | -5 to 2 | -0.43 | 1.50 |
| Walking interference | 0 to 10 | 4.47 | 2.87 | 0 to 9 | 4.61 | 2.51 | **Swollen breasts** | -6 to 2 | **-1.07** | 1.68 |
| Nausea/vomiting | 0 to 10 | 4.64 | 2.34 | 0 to 10 | 4.87 | 2.18 | Fatigue | -4 to 5 | 0.04 | 1.60 |
| Disturbed Sleep | 0 to 10 | 5.05 | 2.67 | 0 to 10 | 5.33 | 2.57 | Nausea/vomiting | -3 to 2 | 0.07 | 1.27 |
| Social contact interference | 0 to 10 | 5.69 | 2.57 | 0 to 10 | 5.50 | 2.78 | Work interference | -3 to 3 | 0.07 | 1.49 |
| Work interference | 0 to 10 | 6.10 | 2.49 | 0 to 10 | 6.37 | 2.12 | Sexual life interference | -2 to 4 | 0.07 | 1.30 |
| Anus bearing-down pain | 0 to 10 | 6.50 | 2.29 | 0 to 10 | 7.09 | 1.81 | Headache | -3 to 6 | 0.11 | 2.01 |
| Sexual life interference | 0 to 10 | 6.65 | 2.16 | 0 to 10 | 7.13 | 1.95 | Frequent urination | -2 to 4 | 0.11 | 1.45 |
| Waist pain | 0 to 10 | 6.67 | 2.25 | 0 to 10 | 7.52 | 1.75 | Walking interference | -5 to 4 | 0.11 | 2.01 |
| Abnormal bearing-down pain | 0 to 10 | 7.33 | 2.16 | 0 to 10 | 7.72 | 1.61 | Abdominal pain | -1 to 2 | 0.21 | 0.63 |
| Mood interference | 0 to 10 | 7.42 | 2.16 | 0 to 10 | 7.46 | 2.22 | Mood interference | -3 to 5 | 0.21 | 1.62 |
| Abdominal pain | 0 to 10 | 8.55 | 1.66 | 0 to 10 | 9.11 | 0.90 | Disturbed Sleep | -5 to 6 | 0.57 | 2.20 |

**Table S3.** Mean Symptom Severity Scores of the Entire Cohort

|  | Mean score (SD) | Missing, n (%) | Score between 0 to 3, n (%) | Score ≥4, n (%) |
| --- | --- | --- | --- | --- |
| Abnormal uterine bleeding | 4.1(3.4) | 11(6.15%) | 75(41.9%) | **93(51.96%)** |
| Abdominal pain | 5.5(3.0) | 7(3.91%) | 48(26.82%) | **124(69.27%)** |
| Waist pain | 3.9(3.1) | 10(5.59%) | 84(46.93%) | **85(47.49%)** |
| Abnormal bearing-down pain | 4.1(2.9) | 9(5.03%) | 82(45.81%) | **88(49.16%)** |
| Anus bearing-down pain | 2.3(2.6) | 12(6.7%) | 122(68.16%) | 45(25.14%) |
| Fatigue | 3.9(3.0) | 10(5.59%) | 86(48.04%) | **83(46.37%)** |
| Dizzy | 2.4(2.9) | 9(5.03%) | 119(66.48%) | 51(28.49%) |
| Disturbed Sleep | 3.4(3.2) | 7(3.91%) | 93(51.96%) | 79(44.13%) |
| Lack of appetite | 2.2(2.9) | 10(5.59%) | 124(69.27%) | 45(25.14%) |
| Frequent urination | 1.8(2.5) | 7(3.91%) | 134(74.86%) | 38(21.23%) |
| Nausea/vomiting | 1.1(2.1) | 9(5.03%) | 149(83.24%) | 21(11.73%) |
| Mood interference | 3.6(3.1) | 10(5.59%) | 97(54.19%) | 72(40.22%) |
| Walking interference | 2.8(3.2) | 13(7.26%) | 106(59.22%) | 60(33.52%) |
| Work interference | 3.6(3.5) | 13(7.26%) | 94(52.51%) | 72(40.22%) |
| Social contact interference | 3.2(3.4) | 12(6.7%) | 102(56.98%) | 65(36.31%) |
| Sexual life interference | 2.3(2.9) | 12(6.7%) | 119(66.48%) | 48(26.82%) |

**Table S4.** Summary statistics for the Rasch analysis (N = 179)

| Statistics | Persons | Items |
| --- | --- | --- |
| Mean Measure | -0.3 | 0.00 |
| Mean SE | 0.39 | 0.18 |
| Separation Index | 1.91 | 4.85 |
| Separation Reliability | 0.81 | 0.96 |

**Table S5.** Rasch item analysis results (N = 179)

| Items | Measure | Error | Infit | | Outfit | | PTMEA |
| --- | --- | --- | --- | --- | --- | --- | --- |
|  |  |  | MnSq | ZStd | MnSq | ZStd | Correlation |
| Abnormal uterine bleeding | -0.12 | 0.03 | 1.62 | 2.2 | 1.75 | 2.2 | 0.65 |
| Abdominal cramping | -0.35 | 0.03 | 0.85 | -1.5 | 0.86 | -1.3 | 0.66 |
| Waist pain | -0.07 | 0.03 | 0.87 | -1.3 | 0.84 | -1.4 | 0.64 |
| Anus bearing-down pain | 0.22 | 0.04 | 1.08 | 0.7 | 0.97 | -0.1 | 0.58 |
| Fatigue | -0.09 | 0.03 | 0.69 | -3.4 | 0.66 | -3.3 | 0.64 |
| Disturbed Sleep | 0.00 | 0.03 | 0.96 | -0.3 | 0.89 | -0.9 | 0.63 |
| Lack of appetite | 0.25 | 0.04 | 1.01 | 0.2 | 0.86 | -0.8 | 0.57 |
| Mood interference | -0.03 | 0.03 | 0.72 | -2.9 | 0.65 | -3.1 | 0.63 |
| Work interference | -0.04 | 0.03 | 1.13 | 1.3 | 1.02 | 0.2 | 0.63 |
| Sexual life interference | 0.22 | 0.04 | 1.42 | 3.2 | 1.32 | 2 | 0.58 |

**Table S6**. Demographic and clinical characteristics of adenomyosis patients in study cross-sectional study.

|  | |  | Cross-sectional study: External validation (n=130) |
| --- | --- | --- | --- |
| Age,y | |  |  |
|  | Mean ± SD | | 42.9±5.6 |
|  | Median (range) | |  |
| BMI (kg/m^2^), mean ± SD | | | 23.5±2.8 |
| Uterine volume, mean ± SD | | | 218.2±111.4 |
| Date of treatment | | |  |
|  | 2017 | | 11(8.5%) |
|  | 2018 | | 21(16.2%) |
|  | 2019 | | 41(31.5%) |
|  | 2020 | | 57(43.8%) |
| Type of adenomyosis | | |  |
|  | Localized | | 64(52%) |
|  | Diffuse | | 59(48%) |
| Anemia |  | |  |
|  | No | | 97(74.6%) |
|  | Yes | | 31(23.8%) |
|  | Unknown | | 2(1.5%) |
| Recurrence |  | |  |
|  | Yes | | 28 (22.6%) |
|  | No | | 54 (43.5%) |
|  | Unknown | | 42 (33.9%) |
| Symptom severity, mean ± SD | | |  |
|  | Abnormal uterine bleeding | | 1.4±2.1 |
|  | Abdominal cramping | | 2.4±2.5 |
|  | Waist pain | | 1.8±2.2 |
|  | Anus bearing-down pain | | 0.9±1.8 |
|  | Fatigue | | 1.1±1.9 |
|  | Disturbed Sleep | | 1.5±2.3 |
|  | Lack of appetite | | 0.3±0.9 |
|  | Mood interference | | 1.0±1.8 |
|  | Work interference | | 0.8±2.0 |
|  | Sexual life interference | | 0.4±1.2 |

**Table S7. Symptom Assessment Scale of Adenomyosis (AM-SAS-10)—(English version)**

**PART 1：****How severe are your menstrual blood loss？**

Patients with adenomyosis have excessive menstruation. We aim to assess the severity of the Menorrhagia you have experienced ***in the last month***. Please select a number from 0 (not present) to 10 (as bad as you can imagine) for each item to indicate the severity

|  | **0 = Not present 10 = As bad as you can imagine** | | | | | | | | | | |
| --- | --- | --- | --- | --- | --- | --- | --- | --- | --- | --- | --- |
|  | 0 | 1 | 2 | 3 | 4 | 5 | 6 | 7 | 8 | 9 | 10 |
| 1. What is the severity of your **Menstrual blood loss** | ○ | ○ | ○ | ○ | ○ | ○ | ○ | ○ | ○ | ○ | ○ |

**PART 2：How severe are your symptoms?**

Patients may experience various symptoms related to the disease or its treatment. We aim to assess the severity of the following symptoms you have experienced ***in the last month***. Please select a number from 0 (not present) to 10 (as bad as you can imagine) for each item to indicate the severity.

|  | **0 = Not present 10 = As bad as you can imagine** | | | | | | | | | | |
| --- | --- | --- | --- | --- | --- | --- | --- | --- | --- | --- | --- |
|  | 0 | 1 | 2 | 3 | 4 | 5 | 6 | 7 | 8 | 9 | 10 |
| 1. What is the severity of your **Abdominal pain**? | ○ | ○ | ○ | ○ | ○ | ○ | ○ | ○ | ○ | ○ | ○ |
| 1. What is the severity of your **Waist pain (soreness)**? | ○ | ○ | ○ | ○ | ○ | ○ | ○ | ○ | ○ | ○ | ○ |
| 1. What is the severity of your **Anus bearing-down pain**? | ○ | ○ | ○ | ○ | ○ | ○ | ○ | ○ | ○ | ○ | ○ |
| 1. What is the severity of your **Fatigue** ? | ○ | ○ | ○ | ○ | ○ | ○ | ○ | ○ | ○ | ○ | ○ |
| 1. What is the severity of your **Disturbed Sleep）**? | ○ | ○ | ○ | ○ | ○ | ○ | ○ | ○ | ○ | ○ | ○ |
| 1. What is the severity of your **Lack of appetite** ? | ○ | ○ | ○ | ○ | ○ | ○ | ○ | ○ | ○ | ○ | ○ |

**PART 3：How severe are your symptoms**

Symptoms frequently interfere with how we feel and function. How much have your symptoms interfered with the following items ***in the last month***:

|  | **0 = Not present 10 = As bad as you can imagine** | | | | | | | | | | |
| --- | --- | --- | --- | --- | --- | --- | --- | --- | --- | --- | --- |
|  | 0 | 1 | 2 | 3 | 4 | 5 | 6 | 7 | 8 | 9 | 10 |
| 1. How much interfered with your **Mood**? | ○ | ○ | ○ | ○ | ○ | ○ | ○ | ○ | ○ | ○ | ○ |
| 1. How much interfered with your **Work**? | ○ | ○ | ○ | ○ | ○ | ○ | ○ | ○ | ○ | ○ | ○ |
| 1. How much interfered with your **Sextual life**？ | ○ | ○ | ○ | ○ | ○ | ○ | ○ | ○ | ○ | ○ | ○ |

**Signature：__________ Date：__________**
